# The relationships between the combination of person- and organization-related conditions and patients’ perceptions of palliative care quality

**DOI:** 10.1186/s12904-017-0240-x

**Published:** 2017-12-06

**Authors:** Tuva Sandsdalen, Sevald Høye, Ingrid Rystedt, Vigdis Abrahamsen Grøndahl, Reidun Hov, Bodil Wilde-Larsson

**Affiliations:** 1 Department of Health Studies, Faculty of Public Health, Inland Norway University of Applied Sciences, Post box 400, 2418 Elverum, Norway; 2 Department of Health Science, Faculty of Health, Science and Technology, Discipline of Nursing Science, Karlstad University, 651 88 Karlstad, Sweden; 3 Faculty of Health and Welfare Sciences, Østfold University College, Post box 700, 1757 Halden, Norway

**Keywords:** Organization, Palliative care, Patient perception, Person-related conditions, Quality of healthcare, Quality from the patients’ perspective specific to palliative care (QPP-PC)

## Abstract

**Background:**

Little is known about the combination of person- and organization- related conditions and the relationships with patients’ perspectives of care quality. Such a combination could contribute knowledge reflecting the complexity of clinical practice, and enhance individualized care. The aim was to investigate the relationships between the combination of person- and organization-related conditions and patients’ perceptions of palliative care quality.

**Methods:**

A cross-sectional study, including 191 patients in the late palliative phase (73% response rate) admitted to hospice inpatient care (*n* = 72), hospice day care (*n* = 51), palliative units in nursing homes (*n* = 30) and home care (*n* = 38), was conducted between November 2013 and December 2014, using the instrument Quality from the Patients’ Perspective specific to palliative care (QPP-PC). Data were analysed, using analysis of covariance, to explore the amount of the variance in the dependent variables (QPP-PC) that could be explained by combination of the independent variables – Person- and organization-related conditions, − while controlling for differences in covariates.

**Results:**

Patients scored the care received and the subjective importance as moderate to high. The combination of person- and organization - related conditions revealed that patients with a high sense of coherence, lower age (person – related conditions) and being in a ward with access to and availability of physicians (organization-related condition) might be associated with significantly higher scores for the quality of care received. Gender (women), daily contact with family and friends, and low health-related quality of life (person-related conditions) might be associated with higher scores for subjective importance of the aspects of care quality.

**Conclusion:**

Healthcare personnel, leaders and policy makers need to pay attention to person- and organization-related conditions in order to provide person-centered palliative care of high quality. Further studies from palliative care contexts are needed to confirm the findings and to investigate additional organizational factors that might influence patients’ perceptions of care quality.

## Background

Patients’ perspectives and their experience of the care received are considered to be important components for service evaluation; the knowledge gained could inform policy-makers, healthcare managers and healthcare personnel how to help identify potential areas for improvement [1, 2]. National health services are required to have systems that systematically collect patients’ experiences of care received [2, 3]. In the palliative care setting, recruitment of patients and obtaining feedback from patients may be difficult [4, 5], because patients have life-threatening illnesses and often have severe physical, psychosocial and existential problems [6], and it may be difficult to locate patients in a palliative phase of illness [7]. Despite these difficulties, it is still important to acquire knowledge about patients’ perceptions of palliative care quality.

Healthcare services aims to provide high-quality care to all patients [2, 3]. What constitutes quality of care in the present study is built on a model developed by Wilde et al. [8]. According to this theoretical framework, the care quality is formed through patient norms, expectations and experience, and an encounter with a care structure [8]. Based on this, care quality can be seen as a measure of patients’ perceptions of the actual care received (the perceived reality) and how important the various care aspects are to them (the subjective importance of the care aspects) [9]. Conditions that influence patients’ perceptions of care quality can be classified into two areas: person-related conditions and organization-related conditions.

### Person-related conditions

Several studies have investigated the relationship between person-related conditions and patients’ perceptions of *care received*. Studies from palliative care settings have found that patients with low levels of education [10], a cancer diagnosis (compared with non-malignant illnesses) [11–13] and multiple medical conditions [14, 15] tend to score the quality of care received as higher. Although some studies have found conflicting results [15, 16], this is supported by previous research from other care contexts [17–23].

The perceptions of care quality received seemed to be associated with higher age [17–19, 24–26], higher psychological wellbeing [24] and higher sense of coherence (SOC) [27], and religious affiliation [28]. These studies were conducted in contexts that were not specifically palliative care.

However, previous research seems to be conflicting or uncertain with regard to the association between patients’ perceptions of care quality received and; gender [18, 21–24], quality of life [10, 16, 20] and physical health [19, 24, 29].

Time in care may be related to patients’ perceptions of care received, as one study found that patients who had been hospitalized for long periods of time rated the care received as higher [22]. However, another study found decreased perception of care received with each additional day in hospital [25].

Patients living alone may experience care differently from patients living with someone [30, 31], but it is not known whether and how this affects their perceptions of care quality. It is well known that family and friends are of important to patients in their late palliative phase of illness [32–34]; however, whether the amount of contact with families and friends is associated with perceptions of care quality is not known.

In relation to patients’ perceptions of care received, few studies have investigated the association between patients’ perceptions of *subjective importance* (SI) and person-related conditions in the palliative care context. Rocker et al. [13] found that patients’ perceptions of subjective importance differed in according to whether patients were diagnosed with cancer or COPD. Studies from settings that are not specifically palliative care contexts the relationships between person-related conditions and perceptions of SI are therefore either uncertain (few studies) or conflicting (contradictory findings) in terms of age [17, 24, 35], gender [23, 24], patients’ SOC [24, 27] and patients physical and psychological wellbeing [23, 24].

Studies that have established associations between patients’ perceptions of SI and education, multiple medical conditions, living conditions, the amount of contact with family and friends, religious affiliations, quality of life or time in care have not been found.

### Organization-related conditions

Patients’ perceptions of *care received* have previously been shown to be associated with ward characteristics such as types of wards, e.g. specialized versus non-specialized [36–38]. However, it is not fully known what constitutes these differences in relation to organizational factors. Previous studies have found that the number of nurses or staff [18, 36, 39] and the qualifications of nurses and physicians [40, 41] are related with perceptions of actual care from patient and healthcare personnel perspectives. Further knowledge is needed of the palliative care context.

Nursing care organized as team nursing or primary nursing did not seemed to affect the perception of care received was ascertained in two studies from a general hospital setting [42, 43]. However, in palliative care it has previously been shown that the continuity of care is of the utmost importance to patients receiving such care [34, 44, 45], so organizational models of both nursing and medical care should enhance continuity. It is therefore important to gain knowledge of how the organization of nursing care and physicians’ medical care affect patients’ perceptions of care received in a palliative care context.

Palliative care includes prevention and relief of physical, psychosocial and spiritual suffering [6], and requires competent healthcare personnel from multiple professions to be available [46], e.g. nurses, physicians, physiotherapists, occupational therapists, nursing assistants, priests, social workers, nutritional physiologists. The availability of multiprofessional staff is important for patients in palliative care [34, 47], so it is important to investigate how their availability relates to patients’ perceptions of care quality received.

Systematic documentation of patient information in general (e.g. individual care plan, patient care records), and assessment tools for systematic assessment and documentation of symptoms, in particular, are necessary to guide care of high quality [46, 48]. Documentation and communication of patient information seem to affect care quality [49, 50], but studies investigating the association between documentation of patient information and patients’ perceptions of actual care in a palliative care context seem to be needed.

Person-centred care (PCC) has been established as an important part of quality of care and involves the acknowledgement of patients’ being the centre of care and being respectful of and responsive to individual patient preferences, needs, and values [48]. In the palliative care context, a person-centred model of care has been developed based on the hospice philosophy of care to enhance care quality [51]. Therefore, it is interesting to investigate whether the use of frameworks that emphasize a set of values (such as PCC or hospice philosophy of care) is associated with patients’ perceptions of care quality. Two reviews have investigated the effect of PCC in relation to satisfaction and perceived quality of care [52, 53]. PCC was beneficially associated with perceived quality of care received, but the results were ambiguous. More studies are needed to support or reject these findings [53].

Studies investigating the relationship between organization-related conditions and *subjective importance* have not been found.

### Combination of person- and organization-related conditions

When investigating the combination of person- and organization-related conditions, person-related conditions (e.g., age, gender, time in care and health condition) has been shown to be the strongest predictor whereas organization-related conditions (e.g., choice of hospital, size of hospital, lengths of stay) to a smaller degree were associated with the patients’ perceptions of *quality of care received* [25, 26]. The impact of person-related conditions, age, gender and psychological wellbeing on patients’ perceptions of care received have been supported in another study from the hospital context, in addition to the organization-related conditions; frequency of over-occupancy and RN headcount [18]. Kirkevold and Engedal [36] found that the care quality in nursing homes from the perspective of the nursing staff were influenced by the person-related conditions; mental capacity, activities of daily living and aggressive behaviour, and the organization-related conditions; type of ward, size of ward and staff to beds ratio influenced the care quality. However, these studies were not performed explicitly in palliative care settings or with patients in the late palliative phase.

Interestingly, studies investigating the relationship between the combination of person- and organization-related conditions and patients’ *perceptions of subjective importance* of the care, in either palliative care or other care settings, have not been found.

To sum up, most previous studies that explored the relationship between perceptions of care quality and personal or organization-related conditions had been conducted in settings other than palliative care. Also most studies investigated the relationship between conditions and *perceptions of care received*, and few explored the relationship between patients’ perceptions of *subjective importance* and person- and organization- related conditions. Little is known about the combination of person- and organization- related conditions and its relationship to patients’ perspectives of palliative care quality. Such a combination may contribute to our knowledge, reflecting the complexity of clinical practice and enhancing individualized care.

The aim was to investigate the relationships between the combination of person- and organization-related conditions and patients’ perceptions of palliative care quality.

## Methods

### Design

A cross-sectional study was conducted. The present study is part of a larger study, which has been published [38, 44].

### Setting

Participants were recruited from settings representing densely and sparsely populated locations in the eastern part of Norway: two inpatient hospices (HICs), two hospice day-care centres (HDs), two palliative units in nursing homes that specialized in palliative care (PUNHs) and two home-care districts (HCs). The HICs and HD settings, and the PUNHs, may be characterized as specializing in palliative care, because these settings exclusively provide palliative care [54]. The home-care settings occasionally providing palliative care may be characterized as non-specialized palliative care services, although both HCs did offer specialized trained personnel, such as palliative care teams and cancer nurses, to patients in a late palliative phase. Palliative care is provided by specialist and community care [46]. Hospice services are services with a special dedication to the hospice philosophy and values, and are organized within community care and specialist care [55]. One of the HICs, the two PUNHs and the two HCs were a part of community care. The other hospice inpatient ward and the day hospices were organized within the specialist healthcare services as palliative care units and day-care centres within a hospital.

### Participants

Patients in a late palliative phase were asked to participate in this present study. The late palliative phase was defined as patients being in an advanced phase of their illness, with a 1-year life expectancy [56–58], and received specialized and non-specialized palliative care services [7]. Inclusion criteria were that patients should; be adult (≥18 years), understand Norwegian, have no cognitive impairment (which was judged by the registered nurse selected as responsible for recruiting patients [RRN]), received care from the services for at least 3 days, and have an advanced, life-threatening illness in a late palliative phase (malignant or non-malignant). This was judged and guided by the RRN’s negative response to the question: ‘Would you be surprised if this patient died within the next year?’ [56]. To aid an RRN’s decision when recruiting patients admitted to non-specialized services (home care), patients’ medical and care records were searched for any indication that the patient was in a palliative phase (e.g. for phrases such as ‘advanced cancer/illness’ or visits by a palliative care team). This was done because home-care services provided care for a variety of patients, not exclusively patients in a late palliative phase. Additionally, patients included in the study should be aware of being in a palliative phase and receiving palliative care (judged by the RRN). The RRN were encouraged to consult with the patients’ physicians and the first author (TS) to discuss any uncertainties that arose about the inclusion criteria and whether or not to include patients in the study.

### Instruments

The instrument Quality from the Patients’ Perspective specific to palliative care (QPP-PC) was used to measure patients’ perceptions of care from the perspective of patients with various life-threatening illnesses who were receiving help from different services [44]. The QPP-PC is based on the theoretical foundation of the validated general instrument QPP [8, 9, 59, 60] and has previously been psychometrically evaluated and described [44]. The instrument consisted of 52 items divided into 4 dimensions representing quality of care: the medical–technical competence of the caregiver (MT; 10 items), the physical–technical conditions of the care organization (PT; 3 items), the identity–oriented approach of the care givers (ID; 20 items) and the sociocultural atmosphere of the care organization (SC; 16 items). Also included were three single items about medical care, personal hygiene and atmosphere in the ward [44].

Each item (e.g. the best possible help for my pain) were measured in two ways; by patients’ experiences of the care received (perceived reality – PR scale) and how important each aspect of care was to the patient (SI scale). PR scale was related to the sentence: ‘This is what I experience …’, and SI scale were related to the sentence: ‘This is how important this is to me …’. A 4-point (Likert-type) response scale, ranging from 1 (do not agree at all) to 4 (fully agree), was used for the PR and SI scales: 1 (of little or no importance) to 4 (of the very highest importance). A non-applicable response alternative was available for each item. A mean value was calculated based on the individual participant’s response to the items in the respective dimension. In the present study Cronbach’s α for the QPP-PC showed α values >0.7 for most dimensions (0.88–0.94), except for the PT dimension where α levels were 0.44 for the PR scale and 0.65 for the SI scale.

*Person- related conditions* comprised 12 conditions: patient characteristics such as age, gender, education, type of diagnosis, number of diagnoses, time in care (the length of patient’s experience with care), living conditions, the amount of contact with family and friends, and religious affiliation, SOC, psychological wellbeing and health-related quality of life.

The SOC scale is a validated scale to measure patients’ life orientation in terms of how people manage stressful situations and stay well [61]; it comprises questions about comprehensibility, manageability and meaningfulness [61, 62]. In the present study the Norwegian 13-item version with a 7-point scale was used [63]. The SOC index was calculated by adding the score from each item, ranging from 13 to 91. High scores represent a strong SOC. Cronbach’s α was 0.78 in the present study.

Physiological wellbeing was measured by one item from the QPP questionnaire [9], related to the sentence: ‘I feel that my physiological wellbeing is …’, using a 5-point Likert type scale ranging from 1 (‘very poor’) to 5 (‘very good’).

Health-related quality of life was measured using the EQ - VAS (one item) from the EQ-5D-3 L questionnaire of the EuroQol group [64, 65]. EQ-5D-3 L is a validated, standardized, generic measure of health [66] [67] used by patients in various countries and settings, and found to be suitable for palliative care [68]. The EQ VAS measures patients’ self-rated health on a vertical, visual analogue scale ranging from 0 to 100, where the endpoints are labelled ‘worst imaginable health state’ (0) and ‘best imaginable health state’ (100).

*Organization-related conditions* included questions related to: setting characteristics (e.g. number of patients a year, number of patients in palliative phase a year, type of diagnosis in the ward), organizational models for nursing and physicians, availability and number of staff (including nurses, cancer nurses, physicians, nursing assistants, physiotherapists, occupational therapists, priests, social workers, nutritional physiologists), personnel’s competence in palliative care (formal education for all available personnel), use of theoretical framework (hospice philosophy and PCC), and use of assessment tools (e.g. ESAS) and documentation (e.g. documentation systems used, individual care plan, frequency of documenting that patients were in late palliative phase).

The selection of person- and organization-related conditions was based on a literature review and the researchers’ knowledge of the field.

### Procedure

The data collection was conducted between November 2013 and December 2014. Patients were included according to the inclusion criteria, asked to participate, and provided with information about the study and how to fill out the questionnaire by the RRN in each setting. The participants could take all the time that they needed to complete the questionnaire and were instructed to return the questionnaire in a sealed envelope; this was stored in the RRN’s office until collection by the researcher. Help with filling out the questionnaire was offered as an interview with one of the researchers (TS). Of the 191 participating patients, 54 were interviewed. Of these, 7 were from HICs, 8 from HDs, 22 from PUNHs and 17 from HCs. The interviews were conducted either in a private room in the ward or in the patients’ homes. Each question in the questionnaire were read aloud to the respondents. To make it easier for some patients, the response scales were enlarged on a separate sheet of paper. Patients answered by pointing at the appropriate response category, and/or they answered verbally. The researcher then wrote their responses in the questionnaire following each question.

Data on organization-related conditions were collected from February to March 2015, and focused on data from the year 2014 (time period for the collection of patient data). For the inpatient and day-care settings, data were obtained from the senior charge nurse of the included wards. For the home-care settings, data were obtained from leaders of home-care services in addition to people who were responsible or had expert knowledge of the field or professions, e.g. the district medical officer who had comprehensive knowledge about the community general practitioners (GPs). All respondents received the questions by mail. The author collected the data through personal contact with each ward, by either visiting the ward in person or receiving the data through a combination of phone and email.

### Analysis

IBM SPSS Statistics Data Editor Software [69], version 23, was used to analyse the data. The descriptive statistics, means and standard deviations, were used to describe person- related conditions, organization- related conditions and patients’ perceptions of quality of care.

Analysis of covariance (ANCOVA) was performed [69, 70] for each QPP-PC dimension on the PR and SI scales and single items, to explore the amount of the variance in the dependent variables that could be explained by a combination of the independent variables, Person- related conditions and organization-related conditions, while controlling for differences in covariate variables.

The dependent variables represented the four dimensions (MT, PT, ID and SC) and the three single items of the QPP-PC for both the PR and the SI scales. Independent variables represented variables categorized as ‘Person-related conditions’ and ‘organization-related conditions’. Person-related conditions comprised categorically independent variables representing patient characteristics (gender, education, type of diagnosis, number of diagnoses, time in care, living conditions, amount of contact with family and friends, religious affiliation and psychological wellbeing). The continuing variables – age, health-related quality of life (EQ VAS) and SOC – were used as covariates.

The independent variables for organization-related conditions originally comprised items about setting characteristics, organizational models for nursing and physicians, availability and number of multiprofessional staff, personnel’s competence in palliative care, use of a theoretical framework, use of assessment tools and documentation. A selection of variables for the final analysis was made based on a research review and the researchers’ knowledge of the field, and an evaluation of: if data were complete; if it was possible to compare data/measure at the same level from the four settings (eight wards); and that the number of variables was adjusted according to the number of settings/wards included. The selection process resulted in four included variables: organizational model for physicians, organizational model for nurses, settings with physicians who have achieved palliative medicine as a subspeciality and systematic assessment of symptoms (ESAS).

The data about organization-related conditions were related to each patient staying in the eight care wards/districts (four settings). The ANCOVA analyses were conducted for each dependent variable with the combination of all independent variables (person- and organization-related conditions), for both PR and SI scales (total of 14 analyses).

Cronbach’s α was performed, for the QPP-PC, at the dimensional level (single items included) of both scales (PR and SI) and for the SOC instrument. The Cronbach’s α values is presented in the description of each instrument and values >0.7 were regarded as desirable [69]. Non-response analysis was performed using independent Student’s *t*-test and χ^2^ test for independence, as appropriate. *P-*value <0.05 was considered statistically significant for all analyses.

## Results

### Description of person- and organization-related conditions and patients’ perceptions of quality of palliative care

A total of 262 patients were asked to participate and 191 returned the questionnaire (response rate = 73%). Of the participants, the mean age was 67 years (standard deviation [SD] = 11.62, range 41–94 years), 57% were female, most had cancer (76%), had a medium-to-high level of education (75%), and 51% were living alone. There were no significant differences between the patients included in the study and those who did not respond (*n* = 71), with regard to age (*P* = 0.569) and gender (*P* = 0.117).

Of the patients that participated, 72 (37%) were recruited from HICs, 51 (27%) from HDs, 30 (16%) from PUNHs and 38 (20%) from HCs. In the wards recruited, most patients met physicians who worked on the wards 153 (80.1%) and were specialists in palliative care 101 (52.9%), models of nursing care were mostly organized as primary nursing 128 (67.0%), and the ESAS was used for most or all patients 150 (78.5%). Person- and organization-related conditions and patients’ perceptions of the care quality are presented in Table 1.

**Table 1:** Person- and organization – related conditions and patients’ perceptions of care quality (*n* = 191)

Person-related conditions	*n* (%)	Missing
Age (years)		7
Mean score (SD)	66.80 (11.62)	
Range	41–94	
Gender		3
Female	108 (57.4)	
Male	80 (42.6)	
Education		5
Compulsory school or equivalent	47 (25.3)	
High school or equivalent	72 (38.7)	
University/university college	67 (36.0)	
Type of diagnosis		1
Malignant illness (cancer)/Mixed malignant and non-malignant illnesses	159 (83.7)	
Non-malignant illness (e.g. COPD, HF, MS, ALS, Parkinson’s disease)	31 (16.3)	
Number of diagnoses		1
One diagnosis	132 (69.5)	
Two or more diagnoses	58 (30.5)	
Living conditions		1
Living alone	97 (51.1)	
Living with a partner/Living with others	82 (43.2)	
Living with children aged <18 years	11 (5.8)	
The amount of contact with family or friends		2
Daily contact	110 (58.2)	
Less than daily contact	79 (41.8)	
Religious affiliation		13
No	93 (52.2)	
Yes	85 (47.8)	
Sense of coherence (SOC total)		44
Mean score (SD)	62.52 (11.06)	
Range	29-91	
Physiological well-being		21
Poor/very poor	20 (11.8)	
Neither good or poor	56 (32.9)	
Good/very good	94 (55.3)	
Health-related quality of life (EQ - VAS)		25
Mean score (SD)	47.92 (20.38)	
Range	0-90	
Time in care		12
3–7 days	32 (17.9)	
8-30 days	52 (29.1)	
31–182 days (1–6 months)	48 (26.8)	
> 183 (6 months)	47 (26.3)	
Organization - related conditions related to each patient		
Organizational model for physicians		0
Physician employed/available in the ward	153 (80.1)	
Physician as patient’s GP in community care	38 (19.9)	
Organizational model for nursing care		0
Team nursing	63 (33.0)	
Primary nursing	128 (67.0)	
Settings with physicians who have achieved palliative medicine as a subspeciality		0
Settings with physicians having subspecialty in palliative medicine	101 (52.9)	
Settings without physicians having subspecialty in palliative medicine	90 (47.1)	
Systematically assessment of symptoms (ESAS)		0
All or most patients	150 (78.5)	
Some or no patients	41 (21.5)	
Patients’ perceptions of palliative care quality		
Perceptions of care received (PR scale) [mean score (SD)]		
Medical–technical competence	3.05 (0.70)	5
Physical–technical conditions	3.50 (0.59)	5
Identity–oriented approach	3.35 (0.50)	2
Sociocultural atmosphere	3.34 (0.52)	6
Medical care (single item)	3.57 (0.73)	5
Personal hygiene (single item)	3.51 (0.70)	61
Atmosphere (single item)	3.89 (0.33)	68*
Perceptions of subjective importance (SI scale) [mean score (SD)]		
Medical–technical competence	3.17 (0.62)	6
Physical–technical conditions	3.49 (0.55)	6
Identity– oriented approach	3.50 (0.47)	3
Sociocultural atmosphere	3.40 (0.49)	5
Medical care (single item)	3.75 (0.53)	5
Personal hygiene (single item)	3.47 (0.71)	54
Atmosphere (single item)	3.79 (0.43)	72*

For categorical variables, *n* (%) is presented. For continuous variables, mean (SD) and range are presented

ALS, amyotrophic lateral sclerosis; COPD, chronic obstructive pulmonary disorder; HF, heart failure; MS, multiple sclerosis

*Including ‘not applicable’ for all home-care patients (*n* = 38)

#### Patients’ perceptions of the care quality

The mean scores of patients’ perceptions of the care received (PR scale) ranged between 3.05 and 3.50 at the dimension level and between 3.51 and 3.89 at the single item level. For patients’ perceptions of subjective importance of care aspects (SI scale), the mean scores ranged from 3.17 to 3.50 at the dimension level and from 3.47 to 3.79 for the single items.

### The relationships between the combination of person- and organization-related conditions and patients’ perceptions of care quality

#### Significant relationships for the PR scale

The relationships between the combined person- and organization-related conditions and each of the four dimensions of patients’ perceptions of perceived reality are presented in Table 2. The results for patients’ perceptions of actual care received (PR scale) showed that the person-related condition ‘sense of coherence’ was statistically significant related to the dimensions: the MT, PT, ID and SC and the single items about medical care (*P*-value = 0.003) and atmosphere (*P*-value = 0.027). In addition, age had a statistically significant relationship to the ID dimension and organizational physician’s model was statistically significant in the single item about medical care (*P*-value = 0.032). This combination of independent variables explained the variance in the dependent variables by: 24% for MT dimension, 22% for PT dimension, 30% for the ID dimension, 28% for the SC dimension; and for single items: 26% for medical care (*R* ^2^ = 0.257) and 29% for atmosphere (*R* ^2^ = 0.285).

**Table 2:** The relationships between the combined person- and organization-related conditions and patients’ perceptions of perceived reality

	Perceived reality							
	Medical–technical competence		Physical–technical conditions		Identity-oriented approach		Sociocultural atmosphere	
	*F* (df, error)	*p*	*F* (df, error)	*p*	*F* (df, error)	*p*	*F* (df, error)	*p*
Person - related conditions								
Age	1.27 (1,99)	0.263	0.07 (1,99)	0.933	4.78 (1,100)	0.031*	2.74 (1,99)	0.101
Gender	0.03 (1,99)	0.857	1.57 (1,99)	0.213	0.03 (1,99)	0.956	3.28 (1,99)	0.073
Education	0.65 (2,99)	0.523	0.91 (2,99)	0.404	0.45 (2,99)	0.642	0.27 (2,99)	0.762
Type of illness	0.35 (1,99)	0.558	0.78 (1,99)	0.378	1.26 (1,99)	0.264	0.17 (1,99)	0.681
Number of illness	0.85 (1,99)	0.359	1.61 (1,99)	0.207	0.04 (1,99)	0.843	0.02 (1,99)	0.883
Time in care	0.29 (3,99)	0.830	0.04 (3,99)	0.988	2.02 (3,99)	0.116	1.38 (3,99)	0.254
Living conditions	0.90 (2,99)	0.409	0.51 (2,99)	0.605	0.95 (2,99)	0.390	0.78 (2,99)	0.462
The amount of contact with family or friends	0.49 (1,99)	0.486	0.03 (1,99)	0.867	3.05 (1,99)	0.084	2.53 (1,99)	0.115
Religious affiliation	0.60 (1,99)	0.440	0.14 (1,99)	0.710	0.07 (1,99)	0.788	0.11 (1,99)	0.738
Sense of coherence	4.47 (1,99)	0.037*	14.92 (1,99)	<0.001*	5.56 (1,100)	0.020*	10.26 (1,100)	0.002*
Physiological wellbeing	0.16 (2,99)	0.855	0.66 (2,99)	0.520	0.74 (2,99)	0.480	1.06 (12,99)	0.352
Health-related quality of life	1.55 (1,99)	0.216	0.44 (1,99)	0.507	1.59 (1,99)	0.210	0.21 (1,99)	0.647
Organization - related conditions								
Organizational model for physicians	0.06 (1,99)	0.811	1.99 (1,99)	0.161	0.25 (1,99)	0.618	3.47 (1,99)	0.065
Organizational model for nurses	0.13 (1,99)	0.716	0.78 (1,99)	0.379	0.09 (1,99)	0.762	1.45 (1,99)	0.232
Subspecialty in palliative medicine (physicians)	1.80 (1,99)	0.183	0.75 (1,99)	0.390	1.79 (1,99)	0.184	0.18 (1,99)	0.673
Systematic assessment of symptoms (ESAS)	0.08 (1,99)	0.775	1.14 (1,99)	0.289	0.01 (1,99)	0.940	0.15 (1,99)	0.699
*R* ^2^	0.243		0.223		0.295		0.278	

*Conditions with significance at *p* – level < 0.05, measured by ANCOVA analysis

#### Direction of the relationships for the PR scale

With regard to patients’ perceptions of care received (PR scale), their SOC was positively related to the dimensions: the MT, PT, ID and SC (Table 3), and the single items about medical care (*B* = 0.021, *t* = 3.079) and atmosphere (*B* = 0.008, *t* = 2.270). The condition ‘age’ was negatively related to the ID dimension (Table 3). Patients who received care from settings where physicians were available on the wards rated the medical care received higher than those who received medical care from GPs (adjusted mean: 3.934 vs 2.957).

**Table 3:** The direction of the significant relationships between the combined person- and organization- related conditions and patients’ perceptions of perceived reality

	Perceived reality											
	Medical–technical competence			Physical–technical conditions			Identity-oriented approach			Socio-cultural atmosphere		
	*B*	*t*	*p*	*B*	*t*	*p*	*B*	*t*	*p*	*B*	*t*	*p*
Person- related conditions												
Age							−0.010	−2.170	0.032			
Sense of coherence	0.015	2.102	0.038	0.025	3.993	<0.001	0.011	2.341	0.021	0.016	3.103	0.002
Organization - related conditions												

Conditions with significance at *p* – level < 0.05, measured by ANCOVA analysis

#### Significant relationships for the SI scale

The relationships between the combined person- and organization-related conditions and each of the four dimensions of patients’ perceptions of subjective importance of care aspects are presented in Table 4. The results for the SI scale showed that the person-related condition gender had a statistically significant relationship with the MT, PT and ID dimensions – and with the single items personal hygiene (*P*-value = 0.036) and atmosphere (*P*-value = 0.013). The person-related condition ‘amount of contact with family and friends’ had a statistically significant relationship with the MT, ID and SC dimensions, and the single item about medical care (*P*- value = 0.29). In addition, the ‘health-related quality of life’ had a statistically significant relationship with the ID dimension. This combination of independent variables explained the variance in the dependent variables by: 18% for the MT dimension, 18% for the PT dimension, 30% for the ID dimension and 24% for the SC dimension; and for the single items: 19% for medical care (*R* ^2^ = 0.187), 24% for personal hygiene (*R* ^2^ = 0.238) and 32% for atmosphere (*R* ^2^ = 0.317).

**Table 4:** The relationships between the combined person- and organization-related conditions and patients’ perceptions of subjective importance

	Subjective importance							
	Medical–technical competence		Physical–technical conditions		Identity-oriented approach		Sociocultural atmosphere	
	*F* (df, error)	*p*	*F* (df, error)	*p*	*F* (df, error)	*p*	*F* (df, error)	*p*
Person - related conditions								
Age	1.00 (1,99)	0.321	0.18 (1,99)	0.671	1.39 (1,100)	0.241	0.26 (1,100)	0.611
Gender	5.63 (1,99)	0.020	4.60 (1,99)	0.035*	5.04 (1,100)	0.027*	3.17 (1,100)	0.078
Education	0.71 (2,99)	0.401	1.35 (2,99)	0.264	0.12 (2,100)	0.897	0.15 (2,100)	0.864
Type of illness	0.02 (1,99)	0.901	0.12 (1,99)	0.730	0.44 (1,100)	0.511	0.04 (1,100)	0.844
Number of illness	0.02 (1,99)	0.890	1.03 (1,99)	0.313	0.15 (1,100)	0.700	0.12 (1,100)	0.729
Time in care	0.28 (3,99)	0.839	1.19 (399)	0.319	1.94 (3,100)	0.128	1.04 (3,100)	0.378
Living conditions	0.03 (2,99)	0.967	0.70 (2,99)	0.500	0.43 (2,100)	0.653	0.19 (2,100)	0.829
The amount of contact with family or friends	5.32 (1, 99)	0.023*	2.79 (1,99)	0.098	11.65 (1,100)	0.001*	12.89 (1,100)	0.001*
Religious affiliation	0.99 (1,99)	0.323	0.07 (1,99)	0.795	0.02 (1,100)	0.887	0.06 (1,100)	0.598
Sense of coherence	2.09 (1,99)	0.151	1.80 (1,99)	0.182	2.02 (1,100)	0.158	0.83 (1,100)	0.336
Physiological wellbeing	0.61 (2,99)	0.544	0.35 (2,99)	0.707	0.14 (2,100)	0.869	0.51 (2,100)	0.603
Health-related quality of life	0.03 (1,99)	0.865	0.97 (1,99)	0.327	4.83 (1,100)	0.030*	1.23 (1,100)	0.271
Organization -related conditions								
Organizational model for doctors	1.34 (1,99)	0.249	0.04 (1,99)	0.853	0.35 (1,100)	0.555	0.95 (1,100)	0.334
Organizational model for nurses	0.03 (1,99)	0.867	0.24 (1,99)	0.624	0.77 (1,100)	0.382	2.19 (1,100)	0.142
Subspecialty in palliative medicine (physicians)	0.71 (1,99)	0.401	0.67 (1,99)	0.417	0.01 (1,100)	0.919	1.84 (1,100)	0.178
Systematic assessment of symptoms (ESAS)	1.44 (1,99)	0.233	0.00 (1,99)	0.952	0.51 (1,100)	0.478	0.49 (1,100)	0.486
*R* ^2^	0.179		0.184		0.296		0.238	

*Conditions with significance at *p* – level < 0.05, measured by ANCOVA analysis

#### Direction of the relationships for the SI scale

For patients’ perceptions of importance of care aspects (SI scale), women scored SI higher than men for three out of four dimensions (adj. Mean: for MT 3.20 vs 2.90; for PT 3.58 vs 3.34; for ID 3.47 vs 3.28) (Table 5), and two single items: hygiene (adj. Mean. 3.90 vs 3.48) and atmosphere (adj. Mean. 3.92 vs 3.61). The SI was also rated higher for patients with daily contact with their families and friends for MT dimension (adj. Mean 3.21 vs 2.90), ID (adj. Mean 3.53 vs 3.22) and SC dimensions (adj. Mean 3.37 vs 3.00) (Table 5), and for the single item about medical care (adj. Mean. 3.84 vs 3.60). The ‘health-related quality of life’ was negatively related to the ID dimension (*B* = −0.005, *t* = −2.198) (Table 5).

**Table 5:** The direction of the significant relationships between the combined person- and organization- related conditions and patients’ perceptions of subjective importance

	Subjective importance											
	Medical–technical competence			Physical–technical conditions			Identity-oriented approach			Socio-cultural atmosphere		
	*B*	*t*	*p*	*B*	*t*	*p*	*B*	*t*	*p*	*B*	*t*	*p*
Person - related conditions												
Gender												
Woman	0.304	2.373	0.020	0.245	2.144	0.035	0.195	2.245	0.023			
Man	0 ^a^			0 ^a^			0 ^a^					
The amount of contact with family or friends												
Daily contact	0.316	2.307	0.023				0.317	3.413	0.001	0.369	3.590	0.001
Less than daily contact	0 ^a^						0 ^a^			0 ^a^		
Health-related quality of life							−0.005	−2.198	0.030			
Organization - related conditions												

Conditions with significance at *p* – level < 0.05, measured by ANCOVA analysis. ^a^Parameter set to zero because it is redundant

## Discussion

Patients’ scores of the perceived reality could be interpreted as that the quality of care received was moderate to high. These mean scores are lower than the scores obtained using the QPP instrument in a Norwegian study including patients in hospitals (not specifically palliative care) [18], but are in line with scores obtained from patients with lung cancer in a Swedish context [24].

Patients’ perceptions of subjective importance of care is lower than in the Swedish study [24]. It is difficult to reach any conclusion based on this, and more studies are needed from the patient perspective in a palliative care context and from the Norwegian context to interpret these results.

The significant association between patients’ perceptions of care received (PR scale) and patients’ SOC, age and availability of a physician on the ward is not fully supported in previous studies. Studies comprising a combination of person- and organization-related conditions, and their relationships with quality of care received, have been conducted including patients who are not specifically in their late palliative phase, from wards in hospitals [18, 25, 26] and nursing homes [36]. The significant impact of age was supported by three out of four of these studies [18, 25, 26], but SOC and availability of physicians were not included in these studies. However, a previous study investigating the association between the person-related condition SOC and quality of care received in wards in the hospital setting [27], found that PR correlated with SOC, especially for the ID and SC dimensions.

The condition ‘age’ was significantly and negatively related to the ID dimension, which means that older patients rated the care received lower than younger patients. This may indicate that older patients in the present study received care of lower quality. However, this finding contradicts findings from studies performed in settings other than palliative care, which more commonly found that elderly patients rated the quality of care received higher than younger patients [17–21, 24–26]. More studies are needed to clarify how age is related to patients’ perceptions of received palliative care.

Patients who received care in settings where physicians were available on the wards rated the care received for the single item about medical care higher than patients receiving medical care from GPs. This may be explained by patients possibly perceiving that access to help from the physicians was more available when on the ward, which led to perceptions of higher quality of medical care received. In the settings included in the present study, care from physicians in hospice inpatient care and day care, as well as palliative units in nursing homes (specialized palliative care), was organized so that physicians were available as part of the staff on a daily basis or for several days a week. For home-care patients, the patients’ GPs are responsible for the medical care (non-specialized palliative care). However, the follow-up and engagement by the GP with patients in the late palliative phases have been found to vary in a recent report evaluating palliative care in Norway [71]. In addition, the reluctance of GPs to make home visits has been pointed out as a challenge for high-quality home care for older patients by the World Health Organization (WHO) [72]. Further studies, including organization of medical care from physicians in the palliative care setting, are needed.

Previous studies including both person- and organization-related conditions and their relationship to SI have not been identified. However, a few studies have investigated the association of person-related factors with SI and these will further be discussed in relation to the findings of this study.

In this present study, women scored SI significantly higher than men for three out of four dimensions (MT, PT, ID) and two single items: hygiene and atmosphere. This finding is in line with one study from medical and surgical hospital wards conducted in four European countries [23], but different from another study that included patients with lung cancer, which found no relationship between gender and SI [24].

The SI was also rated higher for patients with daily contact with their families and friends, for the MT, ID and SC dimensions and the single item about medical care. A previous study, from an advanced home-care setting, found that family members with daily contact with patients rated the SI higher for aspects of care related to MT and ID dimensions, than family members who seldom met the patients [73]. The ratings of family who met patients daily were also more in line with patients’ scores. This study explained the difference in ratings on SI according to contact with family/patients through an increase in the empathy involved in knowing a person well and sharing constantly changing care experiences [73]. In contrast, two studies found that, for patients, with no close relatives or living alone, who receive palliative care, the identity-oriented aspects of care are of the utmost importance [30, 74] compared with the patients living with somebody [30]. It seems that the association between contact with family and patients’ perception of importance is not fully understood and needs further investigation.

The ‘health-related quality of life’ was negatively related to the ID dimension, meaning that patients with a better health-related quality of life scored lower on the importance of the ID dimension. This finding differs from the study of Henoch et al. [24], who found no statistically significant relationships between self-rated physical health and SI of care aspects.

### Methodological discussion

This study has provided the patients’ perspectives of palliative care quality with a high number returning the questionnaire (73%), which is considered to have strengthened the validity of the present study. Those who chose not to respond did not differ with regard to age or gender which reduced the threats of sampling bias [75]. The representativeness [75] was strengthened in the present study by participants with a broad range of time in care and variety of diagnoses being recruited from four different settings, providing both specialized and non-specialized palliative care from both densely and sparsely locations in eastern part of Norway. Even so, not all settings providing palliative care were included, e.g. general units in hospitals and nursing homes. Last, even though patients with a variety of diagnoses were included according to the comprehensive description of the eligible criteria, the results showed that most patients in this study had cancer. The interpretation of the findings must therefore be based on the sample included. A limitation to this study might be that patients’ cognitive status was not formally assesses.

The amount of missing data was relatively high for the SOC scale and the EQ - VAS scale. This may be because these two scales were placed in the final section of the questionnaire. This could indicate that those not responding were the patients with poorest health who were too tired to complete the questionnaire. However, the EQ - VAS scores of the responding patients showed that patients with low health status did participate in this study.

Validated instruments were used to measure quality of palliative care [44]. For the QPP-PC the reliability in this sample was measured using Cronbach’s α, and the α values were above the desired level of 0.7 for most dimensions apart from the dimension PT (PR = 0.44, SI = 0.65). However, this dimension comprised only three items and this may have influenced the low Cronbach’s α value observed [69]. The validity of the QPP-PC instrument is strengthened because the instrument measures both the perception of perceived reality and the subjective importance of the care aspects. This becomes visible in this present study by the results for the SI scores showing that the QPP-PC instrument and the present study address important aspects of care.

The amount of missing data for the two single items in the QPP-PC questionnaire about personal hygiene and atmosphere was high. This was mainly because of the ‘not applicable’ response. For all of the home care patients, the item about the atmosphere was not applicable. For many patients receiving and evaluating day care services, the personal hygiene item was probably not applicable since such help mainly was provided by the home care services before attending day care.

ANCOVA analyses were chosen because most of the independent variables were categorical. Also, by performing ANCOVA, it is possible to control for bias related to differences in the groups and their effects on the results [69], which is considered to be a strength of the present study. In a previously published paper on the same sample, significant differences in patients’ age between the settings were shown [38]. In the analysis, age, SOC and health-related quality of life were taken into consideration and controlled for. The variable “organizational model for physicians” varied according to settings of care. This might have influenced the findings. We can therefore not be sure if the results indicate that it is the specialized palliative care settings that influenced patients’ perceptions of the medical care (single item), or that it was the physicians availability in these wards.

The model for each dimension on the PR and SI scales explained only about 18–30% of the variance, leaving approximately 75% unexplained. It was intended that the present study would include all of the organization-related conditions originally collected in the analysis (see Methods). However, the different services included were organized in very different ways and the services registered data about the services in different ways. This made it difficult to use the data collected in a meaningful way. For example, the home-care districts cared for all patients in need of home care, not just patients in the late palliative phase of their illness. In addition, the home-care districts did not register data about the number of patients in the late palliative phase. This made it impossible to calculate the staff per patient ratio in general and for specific professions, which could be an important measure for the amount of care and multiprofessional care available. However, some of the data were registered in the same way and that made these data possible to be included in the analysis of the present study. Future studies are needed to find better ways to measure the number of staff per patient in the home-care setting for this specific patient group, the availability of multiprofessional care and specialist nursing competence, and further to include these in analysis to gather new knowledge of what influences the patients’ perceptions of palliative care quality. Also, the way services register organization-related conditions needs to be congruent and comparable.

## Conclusion

The result showed that patients’ SOC and age (person-related conditions), and being in a ward with access to and availability of physicians (organization-related condition), were conditions that might be associated with patients’ perceptions of care received. Gender, daily contact with family and friends, and health-related quality of life (person-related conditions) can be associated with the subjective importance that patients perceived their care to be. Although further studies from palliative care contexts are needed to confirm the findings, it is reasonable to suggest that healthcare personnel, leaders and policymakers should pay attention to person- and organization-related conditions in order to provide person-centered palliative care of high quality.

## Abbreviations

ANCOVA: Analysis of covariance
HC: Home care
HDC: Hospice day care
HIC: Hospice inpatient care
ID: Identity-oriented approach dimension
MT: Medical–technical competence dimension
PCC: Person-centred care
PR: Perceived reality
PT: Physical–technical conditions dimension
PUNH: Palliative units in nursing homes
QPP-PC: Quality from the Patients’ Perspective specific to palliative care
RRN: Registered nurse responsible for recruiting patients
SC: Sociocultural atmosphere dimension
SI: Subjective importance
SOC: Sense of coherence
